# Bioimpedance Vector Pattern in Taiwanese and Polish College Students Detected by Bioelectric Impedance Vector Analysis: Preliminary Observations

**DOI:** 10.1100/2012/684865

**Published:** 2012-04-19

**Authors:** Teresa Malecka-Massalska, Agata Smolen, Elzbieta Madro, Wojciech Surtel

**Affiliations:** ^1^Physiology Department, Medical University of Lublin, Radziwiłłowska 11 Street, 20-080 Lublin, Poland; ^2^Department of Mathematics and Biostatistics, Medical University of Lublin, Jaczewskiego 4 Street, 20-090 Lubin, Poland; ^3^Institute of Hematology and Transfusiology, Indiry Gandhi 14 Street, 02-776 Warsaw, Poland; ^4^Department of Electronics, University of Technology, Nadbystrzycka 38a Street, 20-618 Lublin, Poland

## Abstract

*Background and Objectives*. The study was conducted to evaluate soft tissue hydration and mass through pattern analysis of vector plots as height, normalized resistance, and reactance measurements by bioelectric impedance vector analysis in Taiwanese and Polish college students. *Methods*. Whole-body measurements were made with ImpediMed bioimpedance analysis SFB7 BioImp v1.55 (Pinkenba Qld 4008, Australia) in 16 Taiwanese and Polish men and 16 Taiwanese and Polish women. *Results*. Mean vectors of Taiwanese men and women groups versus the Polish men and women groups were characterized by almost the same normalized resistance component with reactance component (separate 95% confidence limits, *P* < 0.05) indicating that there were no differences of soft tissue hydration and mass. *Interpretation and Conclusion*. The evaluation of soft tissue hydration and mass through pattern analysis of vector plots as height, normalized resistance, and reactance measurements by bioelectric impedance vector analysis in Taiwanese and Polish college students did not differ between these two diverse ethnic groups. Further observational research investigating these properties in larger groups would be welcomed to elucidate and/or confirm these findings.

## 1. Introduction

There are many methods for nutritional status assessment. One of them is bioimpedance analysis (BIA) and the assessment of direct bioimpedance measures (resistance (*R*), reactance (*X*_*c*_), and phase angle (PA)). The use of these raw data has gained popularity in nutrition assessment and monitoring of nutrition status in patients [1]. BIA is based on the principle that a fixed, low-voltage, high-frequency alternating current introduced into the human body is conducted almost completely through the fluid compartment of the fat-free mass [2]. BIA evaluates the body components, resistance (*R*) and reactance (*X*_*c*_), by recording a voltage drop in applied current [3]. Resistance is the restriction to the flow of an electric current, primarily related to the amount of water present in the tissues. Reactance is the resistive effect produced by the tissue interfaces and cell membranes [3]. Part of the electrical current is stored by the cell membranes, which acts as capacitors, creating resistive force. It is this reactance that causes the current to lag behind the voltage ultimately creating a phase shift, which is quantified geometrically as the angular transformation of the ratio of reactance to resistance, or PA [4].

BIVA technique is a promising tool, using the pure data obtained by BIA evaluation, for the screening and monitoring of nutrition and hydration status. BIVA has the potential to be used as a routine method at the bedside for assessment and management of body fluids [5]. Bioelectrical impedance vector analysis allows noninvasive evaluation of soft tissue hydration and mass through pattern analysis of vector plots as height, normalized resistance, and reactance measurements [6]. BIVA has been used to allow detection, monitoring, and control of hydration and nutrition status using vector displacement for the feedback on treatment among patients with Alzheimer's disease [7], in stable and nonstable heart failure patients [8], in critically ill and cardiorenal patients [9], in haemodialysis patients [10], in peritoneal dialysis patients [11], and in cancer patients [12].

In healthy populations, BIVA method has been used in modeling the human body shape [13] and monitoring the variation of the hydrate status in healthy term newborns [14].

In particular, raw BIA measurements measured at 50 kHz, because of its reproducibility quality, has been used to determine the differences in soft tissue hydration and mass [6–14].

The aim of our observational study was to perform bioelectrical impedance analysis to investigate whether the position on the *R*-X_c_ plane of impedance vectors from Taiwanese men and women differed from Polish men and women.

## 2. Materials and Methods

### 2.1. Study Design

This observational study investigated whether the position on the *R*-X_c_ plane of impedance vectors from Taiwanese men and women differed from Polish men and women.

### 2.2. Study Populations

Between October 2009 and May 2010, BIA examinations of tissue electrical properties were conducted in sixty-four college students. Thirty-two healthy Taiwanese students were examined: 16 men and 16 women aged between 18 and 40 years old. Thirty-two healthy Polish students were also examined: 16 men and 16 women aged between 20 and 26 years old.

Baseline nutritional assessment was performed by Subjective Global Assessment (SGA). Subjective Global Assessment has been widely used to assess nutritional needs and monitor nutrition therapy in the hospitalized population. It is a simple, safe, and inexpensive test to administer. BIA was performed by a medical doctor using ImpediMed bioimpedance analysis SFB7 BioImp v1.55 (Pinkenba Qld 4008, Australia). Due to previously published research indicating that exercise influences BIA measurements, in particular phase angle, we controlled for this variable in our study. BIA was performed, after a 10-minute rest period. All patients were positioned supine on a bed, with their legs apart and their arms not touching their torso. All evaluations were conducted on the patients' right side by using the 4 surface standard electrode (tetra polar) techniques on the hand and foot. *R*  and *X*_*c*_ were measured directly in ohms at 5, 50, 100, and 200 kHz. *R* and *X*_*c*_ values were measured three times in each patient, and the mean values were used. PA was obtained from the arc-tangent ratio *X*_*c*_ : *R*. To transform the result from radians to degrees, the result that was obtained was multiplied by 180°/*π*. For further analysis, values of *R*, *X*_*c*_, and PA measured at 50 kHz were taken. Fat mass (kg), as a difference between fat-free mass and weight and fat-free mass (kg) derived from total body water divided by the hydration constant were automatically received on the ImpediMed bioimpedance analysis SFB7 BioImp v1.55 equipment.

### 2.3. Bioimpedance

BIA was performed by a medical doctor using ImpediMed bioimpedance analysis SFB7 BioImp v1.55 (Pinkenba Qld 4008, Australia). BIA was performed, after a 10-minute rest period, while the patients were lying supine on a bed, with their legs apart and their arms not touching their torso. All evaluations were conducted on the patients' right side by using the 4 surface standard electrode (tetra polar) technique on the hand and foot. *R* and *X*_*c*_ were measured directly in ohms at 5, 50, 100, 200 kHz. *R* and *X*_*c*_ values were measured three times in each patient, and the mean values were used. PA was obtained from the arc-tangent ratio *X*_*c*_ : *R*. To transform the result from radians to degrees, the result that was obtained was multiplied by 180°/*π*.

### 2.4. Bioelectrical Impedance Vector Analysis

According to the *RX*_*c*_ graph method [15], measurements of *R* and *X*_*c*_ were standardized by the *H* subjects (i.e., *R*/*H* and *X*_*c*_/*H*) and expressed in ohms per meter. By using the bivariate normal distribution of *R*/*H* and *X*_*c*_/*H*, we calculated the bivariate 95% confidence limits for mean impedance vectors of cancer patients and healthy subjects (i.e., the limit containing the magnitude and the phase angle of the mean vectors with 95% probability). Separate 95% confidence limits indicate a statistically significant difference between mean vector positions on the *R*-*X*_*c*_ plane, that is, in their *R*/*H*, *X*_*c*_/*H*, or both components or in their magnitude, phase angle, or both (*P* < 0.05, which is equivalent to a significant Hotelling *t* ^2^ test) [15].

### 2.5. Statistical Methods

Our results are expressed as mean ± SD. The Shapiro-Wilk (S-W) test was used to assess the distribution conformity of examined parameters with a normal distribution; the Fisher (*F*) test was used to assess variance homogeneity. For group comparisons of metric data, we used the Mann-Whitney *U* test. A *P* value < 0.05 was considered statistically significant. The statistical analysis for this study was performed using the computer software STATISTICA v.8.0 (StatSoft, Poland). BIVA was done with BIVA software (version 2002).

## 3. Results

As previously stated, many research studies refer to great reproducibility of direct bioimpedance measurements (*R*, *X*, PA) at 50 kHz. Due to the logic of this reasoning, our *RX*_*c*_ graph method refers to 50 kHz.

Taiwanese and Polish college male and female student's characteristics with average values of protocol variables are reported in Tables 1, 2, 3, and 4.

## 4. Discussion

Phase angle analysis has become a valuable tool to assess health. Even though the biological meaning of PA is not well understood, what is known about PA has awakened new possibilities for its used. It reflects body cell mass and is one of the best markers of cell membrane function because it is related to the ratio between extracellular water and intracellular water. Variations in phase angle values have been reported between women and men, healthy and diseased patients, and in young and older people. Variations in PA have also been noted among individuals at fixed frequency. The observed variations could be due to differences in the capacitive behavior of this group' tissues, associated variability in cell size, membrane permeability, or intracellular composition or associated with differences in the distribution of body fluids among individuals, which may affect the amount of shunting of the current through the interstitial spaces [16].

In healthy populations, there can be considerable differences between phase angle reference values, and these values vary by population. Kyle et al. [17] found that in the Swiss population, PA values were lower (10.5% in men and 7.7% in women) in comparison to the American study sample. Barbosa-Silva et al. [18] also observed similar variations in PA values between gender and age groups. To our knowledge, the lowest values of PA reference values have been found in the German population, in a study by Dittmar [19]. Thus far, there are no phase angle reference values available for healthy Polish and Taiwanese populations. In light of history, there are some shared cultural between the German and Polish populations. German population reference values are probably most closely related to the Polish population.

**Figure 1:**
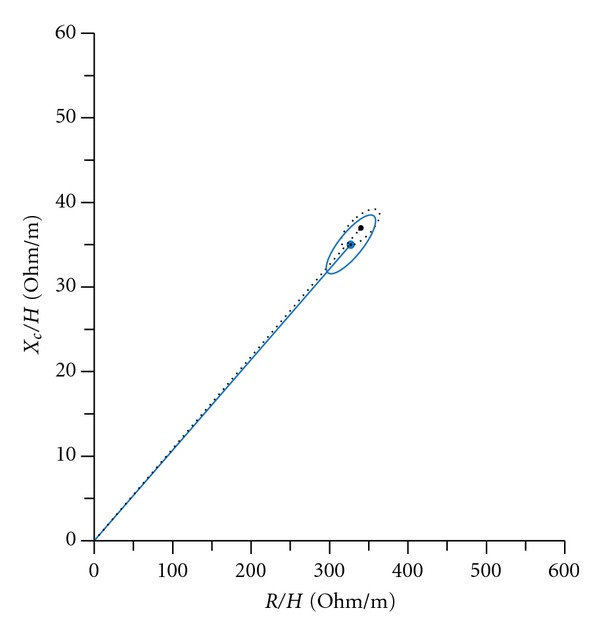
Mean vectors of 95% confidence limits in Taiwanese (black dotted line) and Polish (solid blue line) college men.

**Figure 2:**
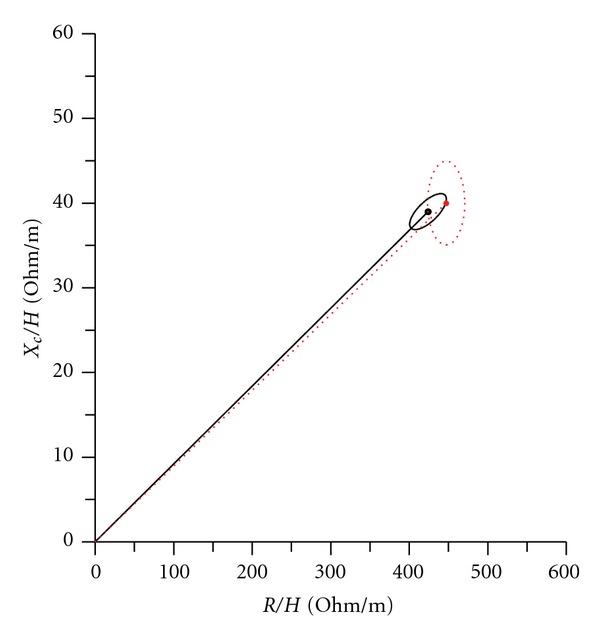
Mean vectors of 95% confidence limits in Taiwanese (red dotted line) and Polish (solid black line) college women.

BIVA allows noninvasive evaluation of soft tissue hydration [6]. In our study, we observed no significant difference vector distribution in either Taiwanese and Polish women or Taiwanese and Polish men by sex and age. The vector displacement of Taiwanese and Polish healthy subjects was characterized by a similar *X*_*c*_ component and similar *R* component [Figures 1 and 2].

Further research is needed to investigate direct bioimpedance measures (resistance, reactance and PA value) on a larger group of Polish and Taiwanese subjects to confirm the similarity of tissue electrical properties for subjects in the age range of our study. This information would also be valuable also in establishing PA reference values for the Polish and Taiwanese populations.

We could consider age to be a factor in our results since the Taiwanese group of students was overall slightly older than the Polish group. However, previous research mentions age as a factor for lower PA due to the influence of exercise or rather, lack of exercise. Since it is shown that as people age, they exercise less [19]. Perhaps because both groups are college students, the activity of college life balanced out any age-related influences.

We cannot understand why we did not observe, as reported by other researchers, statistically significant differences between our student populations. It seems unlikely that these two diverse ethnic groups' results would have such similar PA, resistance, and reactance values.

## 5. Conclusion

In conclusion, our study did not indicate any statistically significant differences in electrical tissue properties, expressed by resistance, reactance, and phase angle between Taiwanese and Polish college students. Mean vectors of Taiwanese men and women groups versus the Polish men and women groups were characterized by almost the same normalized resistance component with reactance component. We observed no statistically significant differences either at different frequencies, among women, men, and/or overall. Further observational research investigating these properties in larger groups would be welcomed to elucidate and/or confirm these findings.

## Figures and Tables

**Table 1:** Baseline characteristics of the Taiwanese and Polish college male students^1^.

Characteristic	Value (Taiwanese men)	Value (Polish men)	*P*
Age (y)	25.81 ± 5.5	21.13 ± 1.54	**0.0001**
BMI kg/m^2^	23.06 ± 2.59	23.09 ± 2.81	0.9
Height (cm)	171.5 ± 7.32	179.13 ± 6.18	0.95
Weight (kg)	68.13 ± 10.81	74.34 ± 11.80	0.92
*R* at 50 kHz (ohm)	582.44 ± 59.32	584.38 ± 68.42	0.19
*R*/*H* (ohm/m)	340 ± 34.86	327.34 ± 43.88	0.15
*X*_*c*_ at 50 kHz (ohm)	63.38 ± 4.88	62.9 ± 7.51	0.71
*X*_*c*_/*H* (ohm/m)	37.03 ± 3.36	35.22 ± 5.03	0.35
Phase angle at 50 kHz (°)	6.23 ± 0.43	6.16 ± 0.5	0.85

^1^ *n* = 16; ±SD; range in parentheses (all such values).

**Table 2:** Electrical tissue properties characteristics of the Taiwanese and Polish college male students^1^.

Characteristic	Value (Taiwanese college male students)	Value (Polish college male students)
Resistance at 5 kHz (ohm) Reactance at 5 kHz (ohm) Phase angle at 5 kHz (°)	673.8 ± 63.08 34.24 ± 3.54 2.93 ± 0.3	676.21 ± 75.41 34.26 ± 3.81 2.93 ± 0.34
Resistance at 100 kHz (ohm) Reactance at 100 kHz (ohm) Phase angle at 100 kHz (°)	552.08 ± 57.33 52.96 ± 4.95 5.51 ± 0.46	554.32 ± 65.55 52.71 ± 6.10 5.45 ± 0.46
Resistance at 200 kHz (ohm) Reactance at 200 kHz (ohm) Phase angle at 200 kHz (°)	526.88 ± 56.78 47.2 ± 5.67 5.13 ± 0.53	528.76 ± 62.88 45.61 ± 5.7 4.93 ± 0.31

^1^ *n* = 16; ±SD; range in parentheses (all such values).

**Table 3:** Baseline characteristics of the Taiwanese and Polish college female students^1^.

Characteristic	Value (Taiwanese men)	Value (Polish men)	*P*
Age (y)	25.81 ± 5.5	21.13 ± 1.54	**0.000001**
BMI kg/m^2^	23.06 ± 2.59	23.09 ± 2.81	0.4
Height (cm)	171.5 ± 7.32	179.13 ± 6.18	0.5
Weight (kg)	68.13 ± 10.81	74.34 ± 11.80	0.26
*R* at 50 kHz (ohm)	582.44 ± 59.32	584.38 ± 68.42	0.59
*R*/*H* (ohm/m)	340 ± 34.86	327.34 ± 43.88	0.47
*X*_*c*_ at 50 kHz (ohm)	63.38 ± 4.88	62.9 ± 7.51	0.72
*X*_*c*_/*H* (ohm/m)	37.03 ± 3.36	35.22 ± 5.03	0.84
Phase angle at 50 kHz (°)	6.23 ± 0.43	6.16 ± 0.5	0.47

**Table 4:** Electrical tissue properties characteristics of the Taiwanese and Polish college female students^1^.

Characteristic	Value (Taiwanese college female students)	Value (Polish college female students)
Resistance at 5 kHz (ohm) Reactance at 5 kHz (ohm) Phase angle at 5 kHz (°)	799.24 ± 49.79 34.26 ± 12.16 2.48 ± 0.98	787.25 ± 46.78 32.11 ± 3.16 2.34 ± 0.25
Resistance at 100 kHz (ohm) Reactance at 100 kHz (ohm) Phase angle at 100 kHz (°)	681.91 ± 54.14 55.19 ± 9.03 4.68 ± 0.9	669.28 ± 42.93 56.5 ± 4.63 4.84 ± 0.38
Resistance at 200 kHz (ohm) Reactance at 200 kHz (ohm) Phase angle at 200 kHz (°)	654.64 ± 53.95 52.88 ± 6.04 4.65 ± 0.68	642.11 ± 42.74 49.23 ± 5.98 4.39 ± 0.4

^1^ *n* = 16; ±SD; range in parentheses (all such values).
